# Translation and validation of health professionals’ knowledge and attitudes instrument regarding Baby-Led Weaning approach into European Portuguese

**DOI:** 10.1177/22799036251369405

**Published:** 2025-10-08

**Authors:** Paula Sarreira-de-Oliveira, Renata Ramalho, Ricardo Antunes, Fernanda Loureiro

**Affiliations:** 1 Egas Moniz School of Health and Science, Egas Moniz Center for Interdisciplinary Research, (CiiEM), Campus Universitário, Quinta da Granja, Monte de Caparica, Caparica, Portugal; 2 Egas Moniz School of Health and Science, Nutritional Immunology – Clinical and Experimental Lab (NICE Lab), Clinical Research Unit, Egas Moniz Center for Interdisciplinary Research (CiiEM), Campus Universitário, Quinta da Granja, Monte de Caparica, Caparica, Portugal; 3 Egas Moniz School of Health and Science, Egas Moniz Center for Interdisciplinary Research (CiiEM), Campus Universitário, Quinta da Granja, Monte de Caparica, Caparica, Portugal; 4 Universidade Católica Portuguesa, Faculdade de Ciências da Saúde e Enfermagem, Centro de Investigação Interdisciplinar em Saúde (CIIS), Palma de Cima, Lisboa, Portugal

**Keywords:** Baby-Led Weaning, health care professional, knowledge, attitudes, infant nutrition

## Abstract

**Background:**

Early childhood is a critical period for preventing malnutrition and ensuring adequate nutrient intake. While various feeding approaches exist, no consensus has been reached on the ideal one. Baby-Led Weaning (BLW) is an emerging method that involves the direct introduction of finger foods to children. However, limited evidence exists on health professionals’ (HP) perspective. We aim to translate and validate an instrument to assess the knowledge and attitudes of HP toward BLW method in European Portuguese.

**Design and methods:**

A methodological study was conducted with a nonprobability sample of 118 HPs working in primary care settings. The instrument underwent translation, back-translation, and review by an expert panel to ensure semantic and conceptual equivalence. The content validity index (CVI) and exploratory factor analysis (EFA) were employed to assess content and construct validity, respectively. Internal consistency was measured using Cronbach’s alpha. The instrument was available online.

**Results:**

The adapted instrument showed high content validity, with CVI values of 0.89 for practice-related items and 0.87 for knowledge and attitude items. EFA revealed three distinct factors: positive child-centered contributions, positive parent-centered contributions, and BLW constraints, explaining 67.46% of total variance. The model’s suitability was confirmed by Bartlett’s test of sphericity (χ²(66) = 665.735; *p* < 0.001) and the KMO index (0.829). All dimensions demonstrated good internal consistency (Cronbach’s α > 0.7).

**Conclusions:**

The validated instrument is a reliable tool for assessing HPs’ knowledge and attitudes toward BLW. Findings support the need for targeted education to better equip professionals in guiding parents on BLW practices.

## Background

The first years of life are crucial for preventing malnutrition and ensuring adequate intake of nutrients.^1^ There is no consensus on the ideal pattern for food introduction, although there are various proposed schemes.^2^ Complementary feeding is defined as the inclusion of solid and liquid foods other than breast milk or infant formula.^2^ Traditionally, parents have spoon-fed their children with fixed recommended portions however, in recent years, there has been a trend toward a comprehensive approach to introducing new foods to children, starting with finger foods, known as Baby-Led Weaning (BLW).^3–5^ Despite this growing trend among parents and researchers, as evidenced by the increasing number of studies in the scientific literature, there are still important unanswered questions about BLW.^6^

There is a paucity of studies on the use and impact of the BLW on children’s health, and even fewer studies have evaluated the health professionals’ perspectives on this method. Health care professionals appear to have some knowledge or familiarity with BLW according to research performed in New Zealand,^7^ Spain,^8,9^ and Brazil,^10^ although the need for additional research on this topic is emphasized in all these studies.

It is important to specify what is meant by knowledge and attitudes when trying to understand what health professionals know and do about BLW. In this context, knowledge is what health professionals know about the subject based on what they have studied or experienced. The concept of attitude is frequently used but often poorly or vaguely defined.^11^ Attitudes, which are predispositions to respond to stimuli, can be cognitive, affective, or behavioral. They are important to study as they influence behavior, perception, and memory.^12^

The development of research and its comparison with other realities is a way to increase the knowledge for which valid tools are necessary. In fact, for health, social and behavioral science research, the development and validation of instruments is critical. An instrument is typically used to assess a behavior, an attitude, or an action that cannot be measured by a single variable or a single item. The use of several items to assess an underlying latent construct can further account for and separate item-specific measurement errors, resulting in more accurate research findings.^13^ Assessment and quantification occur every day and enhance the ability to make good decisions that can impact children and families, quality of life, and quality of care. The true value of any health assessment depends on the strength and likelihood of the instrument measuring it.^14^

A structured BLW instrument was developed by Rubio^8^ in Spain. This study aimed to assess the approach to the introduction of complementary feeding and familiarity with the BLW method by health professionals. Later, the instrument was adapted and used in two other studies, one in Spain^9,15^ and one in Brazil.^10^ In this study, we aimed to adapt and validate this instrument to assess the knowledge and attitudes of health professionals about BLW to European Portuguese.

## Method

This was a methodological study developed with a nonprobability sample of 180 health professionals working in primary care at the Region of Lisbon and Tejo Vale Area, in Portugal. The reporting of this study conforms to the recommendations for studies of instrument and scale development and testing,^16^ as the most appropriate framework available for reporting translation and validation procedures.

First, we conducted a literature review aimed at identifying the literature on health professionals’ knowledge, perceptions and attitudes toward BLW.^17^ We identified a structured instrument with 25 closed-ended questions regarding the approach to introducing complementary feeding and assessing familiarity with the BLW method.^18^ It includes three parts: eight questions addressing health professionals’ practices; 12 questions focusing on their knowledge and attitudes; and five questions regarding socio-demographic data. This first version of the instrument was applied to pediatricians in Spain. Later, Arias-Ramos et al.^9^ adapted the instrument and applied it to health care professionals (doctors, nurses, and midwives) also in Spain. Neves et al.^10^ used the same instrument to describe Brazilian health professionals’ (nursing, speech therapy, medicine, nutrition, and dentistry) perceptions of the use of the BLW method for complementary feeding. However, this study only involved a simple translation of the instrument and did not evaluate its psychometric properties. These properties were not assessed in any of the above studies either.

Initially, in October 2023, we contacted both the authors of the original instrument^18^ and the authors of the Brazilian version.^10^ After obtaining their agreement, we started the process of translation.

We followed the recommendations for the cross-cultural adaptation of health status measures.^19^ We started with the original instrument and sent it to two translators, both of whom were fluent native Spanish speakers and who produced two translations (T1 and T2). One person was a health professional who was knowledgeable about the topic, while the other person was a non-health professional with no particular knowledge of the topic. A consensus version was then created by the two translators and two members of the research team (T12).

The next step was to send this version (T12) to two different translators for back translation (BT1 and BT2). Both were fluent in Spanish and had no knowledge of the original instrument. The same approach was used: one person was a health professional with some expertise in the field, while the other translator had no prior knowledge. In the next step, all the produced versions of the instrument (T1, T2, T12, BT1, and BT2) were sent to an expert panel composed of two methodologists with experience in the translation/adaptation of instruments, two health professionals and two translators with Spanish as their native language. The goal was to establish semantic and conceptual equivalence, and a pretest version was created. To assess content validity, the Content Validity Index (CVI) was used as an indicator of expert consensus, with a value of 0.90 considered optimal.^20^ The translated version of this questionnaire consisted of two parts. The first part included eight sentences characterizing practices related to BLW. The CVI ranged between 0.67 and 1, with a mean value of 0.89. The second part included 12 sentences concerning health professionals’ knowledge and attitudes toward BLW and CIV and ranged from 0.83 to 1, with a mean value of 0.87. For items with lower CVI values, the translations were the subject of discussion until a consensus translation was reached.

To test the pretest version literature recommends using sample size of 30 participants.^21^ In our study, the pretest version of the instrument was administered to a sample of 40 primary care healthcare professionals which represents 34% of our final sample. Because we wanted to apply the study in a specific geographical area, we administered the pretest in a separate area and these responses were not considered in the study. We used the same inclusion criteria as did the original authors of the instrument: health professionals who worked in primary care, were directly involved in child nutrition, and were familiar with the BLW method. There was no need to adapt the instrument, so the pretest version was retained. The instrument was administered to health professionals working in the Lisbon and Tejo Valley region. In Portugal, professionals typically involved in child nutrition are doctors and nurses during routine child health surveillance consultations. However, in certain contexts where nutritionists are part of the care team, they may also play a role in child nutrition. Therefore, the instrument was made available to all health professionals who met the inclusion criteria, regardless of their specific profession.

For the determination of psychometric properties, it is appropriate to define in advance the sample size, which should be in the range of 2 to 20 valid responses for each variable.^22^ Although the instrument is composed of 25 questions, only 12 questions specifically address the knowledge and attitudes of health professionals. Therefore, we defined five responses per item, resulting in a desired sample size of >60. For the data analysis, IBM SPSS Statistics, version 28.0 (2021) was used. Principal component analysis with varimax rotation was used to measure the construct validity of the instrument. Bartlett’s test of sphericity (BTS < 0.05), the Kaiser and Rice method and the Kaiser-Meyer-Olkin tests (with a minimum acceptable KMO value > 0.6) were used to ensure the suitability of the factor model for the correlation matrix and subsequent factor analysis.^23^ The components were retained based on the criteria of eigenvalues greater than 1 and graphical examination of the scree plot. Cronbach’s alpha coefficient was used to measure internal consistency. This measure reduces the amount of data by evaluating the degree of homogeneity or consistency among the original variables, thereby identifying a smaller set of hypothetical variables or components that can be considered latent dimensions.^20^

This study was approved by the Technical and Scientific Council of Egas Moniz on February 1, 2023, and by the Ethics Committee of Egas Moniz on March 30, 2023 (approval no. 1213.23). Since the study was applied in the Region of Lisbon and Tejo Vale Area, it was also submitted to the Ethics Committee of this institution. Approval was granted on September 15, 2023 (approval 50297/2023). An explanation of the study and the intended nature of the participation were included in the online questionnaire. It stated that participation was voluntary, and the health care professional could withdraw from the study at any time. We also ensured anonymity, confidentiality, and data protection. Informed consent was obtained prior to participation. Health professionals could only enroll in the study by clicking a button to indicate their agreement to participate. The instrument was applied over a period of 3 months between October and December 2024.

## Results

In the first phase, the instrument was administered to 40 health professionals, and only two provided any form of comment. One mentioned that the introduction and the ethical aspects were too long. We reformulated our text to make it easier to read. The other comment concerned the organization of the questionnaire, and we also changed it accordingly. Since none of the health professionals commented on the questions themselves, we considered them to be clear and understandable.

In the second phase, the questionnaire was disseminated through a professional email list, and the initial sample consisted of 180 healthcare professionals. To ensure that the inclusion criteria were met, the first question on the questionnaire was “are you directly or indirectly involved in child nutrition? (e.g. by conducting consultations for child health surveillance).” Based on this question, 31 health professionals were excluded from our initial sample (*n* = 149), as shown in Table 1.

**Table 1.** Sociodemographic characteristics of the sample.

Variables	*n*	%
Gender		
Male	21	14.1%
Female	128	85.9%
Age		
23–39 years	30	20.1%
40–49 years	53	35.6%
≥50 years	66	44.3%
Occupation		
Nurse	124	83.2%
Nutritionist/Dietitian	16	10.7%
Other health professional	9	6%

The second question asked whether participants were familiar with the BLW approach. Thirty-one health professionals responded “no” and were subsequently directed to complete only the sociodemographic section. The remaining 118 participants proceeded to answer the full questionnaire.

Exploratory factor analysis (EFA) of the correlation matrix was used to analyze the psychometric properties of the relational structure of the responses to the 12 questions. Principal component analysis followed by varimax rotation was used to extract the factors. The desired number of factors was not predetermined. The three factors were extracted from the components with eigenvalues greater than 1 and in accordance with the analysis of the scree plot. The percentage of retained variance was 67.46%. The validity of the instrument was assessed using Bartlett’s test of sphericity (χ² (66) = 665.735; *p* < 0.001) and the Kaiser–Meyer–Olkin measure of sampling adequacy (KMO = 0.829). The factor loadings of each item on each of the three factors, the eigenvalues, the item communality, and the percent variance explained by each factor are presented in Table 2.

**Table 2.** Matrix of item loadings in the principal component factor analysis with varimax orthogonal rotation.

Dimensions	Rotation matrix	Components			h^2^
	Abbreviated item	1	2	3	
Positive child-centered contributions	BLW can contribute to children’s development	0.838			0.721
	BLW promotes the development of fine motor skills	0.827			0.717
	BLW enhances chewing instead of sucking	0.791			0.674
	BLW eases transition to family meals	0.763			0.679
	BLW can make it easier for children to adapt to the tastes and consistencies of food	0.676			0.577
	BLW can prevent obesity	0.577			0.672
Positive parent-centered contributions	BLW can generate less worry or anxiety for parents/caregivers		0.834		0.699
	BLW helps mothers simplify food introduction		0.771		0.707
	BLW can prevent food-related conflicts		0.695		0.630
	BLW can be very convenient, as there is no need to prepare special food for children (as it simplifies food preparation)		0.386		0.395
BLW constraints	BLW babies will not gain enough weight			0.890	0.832
	BLW can contribute to a lack of certain nutrients			0.881	0.792
Total variance	67.46%				
Variance explained by component		41.79%	15.97%	9.71%	
Eigenvalue		5.012	1.917	1.166	

h^2^ = communalities.

Table 2 shows the total items retained and the three dimensions that account for 6.46% of the variance explained. The item loadings in the first dimension ranged from 0.838 to 0.577, in the second dimension from 0.834 to 0.386, and in the third dimension from 0.890 to 0.881. The communality values (h^2^) were greater than 50% for all items except for “BLW can be very convenient as there is no need to prepare special food for children (as it simplifies food preparation)”; however, we decided to keep this as it is pointed out in the literature as one of the main advantages of this method.^24,25^ In addition, the interpretation of the items was combined with the designation of health professionals’ knowledge and attitudes on BLW in the three final dimensions proposed for the Portuguese version of the instrument. Cronbach’s alpha was calculated for both the total instrument and each of the three dimensions to measure internal consistency. The total scale had a value of 0.790. Given the good Cronbach’s alpha values obtained, the arithmetic mean was calculated separately for each dimension, as detailed in Table 3.

**Table 3.** Dimensions and means of health professionals’ knowledge and attitudes regarding BLW.

Dimensions	Cronbach’s alpha	Mean
Positive child-centered contributions	0.881	4.19
Positive parent-centered contributions	0.719	3.61
BLW constraints	0.810	2.83

## Discussion

The aim of our study was to adapt and validate an instrument to assess the knowledge and attitudes of health professionals toward BLW in European Portuguese. Given the increasing adoption of the BLW method by parents and the influence that health care professionals have on this choice, validation of this instrument is a critical step in accurately assessing the attitudes and knowledge of health care professionals.

We followed well-established guidelines for translating and adapting an instrument, which ensured a systematic and scientifically rigorous process, as recommended.^26^ This included translation, back-translation, and review by an expert panel to ensure semantic and conceptual equivalence. To support the validity of a measurement instrument, content validity—the degree to which a measurement instrument accurately captures the construct being measured—must be present.^20^ The CVI is used to assess content validity, and considering the number of experts used (6), the acceptable cutoff score of the CVI should be at least 0.83.^27^ Our study achieved an average score of 0.89, which is a positive finding and in line with the recommendations.

With respect to psychometric evaluation, we used a traditional approach to assess reliability and validity.^28^ Given that this is a new instrument in its early stages of development, it is recommended that EFA be conducted in three steps: data suitability assessment, factor extraction, and factor rotation and interpretation.^29^ The suitability of the data was assessed by means of two statistical tests (Bartlett’s test of sphericity and the Kaiser–Meyer–Olkin measure of sampling adequacy). Regarding factor extraction, the Kaiser criterion and scree plot were used. Finally, for factor rotation and interpretation, varimax rotation was used. This process allowed us to identify three main factors that explained 67.46% of the total variance. Because no previous studies using this instrument have assessed psychometric properties, comparisons are not possible. Nevertheless, we have adhered to a widely recognized methodological procedure that reliably assures the validity of the process.

With respect to internal reliability, as measured by Cronbach’s alpha coefficient, the results were considered good, with values above 0.7 for all dimensions and for the total scale (α = 0.790). These values indicate a high degree of internal consistency, suggesting that the items within each dimension are coherent and effectively measure the underlying construct. This is an important finding given that the instrument was recently developed.

The results revealed three dimensions of health professionals’ knowledge and attitudes: positive child-centered contributions, positive parent-centered contributions, and BLW constraints. These dimensions are consistent with the literature on the implementation of BLW, which highlights its benefits both from the perspectives of children and parents and from the perspectives of health professionals.^30,31^

The first dimension, positive child-centered contributions, includes benefits such as promoting fine motor skills, facilitating the transition to family meals, and supporting obesity prevention. These findings align with several advantages reported in the existing literature^6^ and suggest that BLW may facilitate the acceptance of different textures and tastes, thereby promoting healthier eating habits in the long term.

The second dimension, positive parent-centered contributions, highlights the positive impact of parental factors, for example, convenience and reduction in parental anxiety associated with BLW. Health professionals perceive BLW as an approach that simplifies food introduction and minimizes food-related conflicts.^32^ These aspects can be particularly important for parents seeking practical and less stressful methods to introduce solid foods. Although the literature points to some benefits from the parents’ perspective, it also highlights the parents’ difficulties and the lack of concrete guidance on how to implement the BLW method.^33^ In traditional method, one may consider that it may be easier for families to introduce children to food since it is prepared for the entire family. However, we must acknowledge whether this approach respects children’s needs regarding, for example, their sodium intake.^34^

The third dimension, BLW constraints, repeatedly appears in the literature as a barrier to the implementation of the BLW method.^30,31^ These constraints include nutritional concerns,^25^ informational sources,^9^ and even cultural barriers.^4^ However, the authors note that there is not enough data to support these perceived constraints. More recently, Fernández-Medina et al.^32^ concluded that healthcare professionals perceived BLW as a safe and natural approach to weaning. The same author, however, also pointed that the lack of training of health professionals and the social context of the family could have decisive influences on the implementation of the BLW method. It is important to note, however, that there is still insufficient conclusive evidence linking BLW with improved growth outcomes and adequate nutrient intake and therefore BLW is not currently recommended over a traditional approach.^30^

By validating this instrument to assess knowledge and attitudes, we are creating a pathway for further research and to better support parents considering BLW for their children. The adaptation and validation of this instrument allows for a more accurate assessment of the level of knowledge and attitudes of health professionals, facilitating the identification of specific areas requiring educational intervention.

This study has limitations that should be acknowledged. Comparable data are not available to contextualize our findings or to assess the consistency of psychometric properties across samples and settings because the instrument used has not been validated in other studies. Although the sample size of this study falls within the generally recommended range for conducting EFA, a larger and more diverse sample would have strengthened the reliability and generalizability of the results. This sample size limited the statistical analyses and the ability to detect more nuanced patterns or relationships.

The exclusion of participants who reported unfamiliarity with the BLW approach resulted in a reduction of responses considered in the psychometric analysis to 118. Although this decision was methodologically justified, since unfamiliar respondents could not meaningfully answer questions on the topic, it nevertheless limited the scope of the analysis and may introduce a form of selection bias. This limitation may potentially impact the generalizability of the findings to a broader population of health professionals, including those with less experience with BLW. As with any self-report questionnaire, the possibility of social desirability bias exists. That is, participants may provide answers they perceive as more professionally acceptable, rather than reflecting their true beliefs or practices.

Future research should aim to validate this instrument in larger and more varied populations, explore its use in different cultural and professional contexts, and assess its test–retest reliability and convergent validity with other established measures.

## Conclusion

The adaptation and validation of the instrument to assess health professionals’ knowledge and attitudes about BLW in European Portuguese revealed a robust factor structure and adequate internal reliability. These results highlight the importance of a validated instrument for the accurate assessment of health professionals’ perceptions, facilitating targeted educational interventions and supporting the safe and effective implementation of BLW. Continued research and ongoing education are essential to ensure that health professionals can competently support parents who choose this complementary feeding approach.

## Supplemental Material

sj-docx-1-phj-10.1177_22799036251369405 – Supplemental material for Translation and validation of health professionals’ knowledge and attitudes instrument regarding Baby-Led Weaning approach into European Portuguese Supplemental material, sj-docx-1-phj-10.1177_22799036251369405 for Translation and validation of health professionals’ knowledge and attitudes instrument regarding Baby-Led Weaning approach into European Portuguese by Paula Sarreira-de-Oliveira, Renata Ramalho, Ricardo Antunes and Fernanda Loureiro in Journal of Public Health Research
