# Supplementary material for: Translation and validation of health professionals’ knowledge and attitudes instrument regarding Baby-Led Weaning approach into European Portuguese
Source: J Public Health Res. 2025 Oct 8;14(4):22799036251369405. doi: 10.1177/22799036251369405 (PMC12511716; doi:10.1177/22799036251369405)
Supplement: sj-docx-1-phj-10.1177_22799036251369405 – Supplemental material for Translation and validation of health professionals’ knowledge and attitudes instrument regarding Baby-Led Weaning approach into European Portuguese [file sj-docx-1-phj-10.1177_22799036251369405.docx]

**Supplemental File 1**

**Health professionals' knowledge and attitudes towards the Baby-Led Weaning instrument**

1. Are you directly or indirectly involved in child nutrition? (e.g., by conducting consultations for child health surveillance)
   1. Yes (go to question 2)
   2. No (excluded)
2. Are you familiar with the Baby Led Weaning approach?
   1. Yes (go to question 3)
   2. No (excluded)
3. What was your main source of information about the BLW method?
   1. The children's own parents / carers
   2. Training (course / lecture / meeting / conference etc.)
   3. Website
   4. Other health professionals
4. Do you know the benefits of the BLW method?
   1. Yes
   2. No
5. Do you recommend practicing the BLW method?
   1. Always (go to question 7)
   2. Frequently (go to question 7)
   3. Sometimes (go to question 7)
   4. Never (go to question 6)
6. What's the main reason you never recommend the BLW method?
   1. Fear that the child may suffocate
   2. I'm afraid the BLW method leads to insufficient nutritional intake
   3. Risk of iron deficiency/anaemia
   4. Scarcity of scientific evidence
   5. Insufficient information
7. Do you currently have families using this method?
   1. Yes
   2. No
8. Have you followed or do you follow any children / families who use the BLW method?
   1. Yes
   2. No
9. Knowledge of the Baby Led Weaning (BLW) method
   1. BLW eases transition to family meals (I totally agree / Partially agree / Indifferent / Partially disagree / Totally disagree)
   2. BLW can generate less worry or anxiety for parents/caregivers (I totally agree / Partially agree / Indifferent / Partially disagree / Totally disagree)
   3. BLW can make it easier for children to adapt to the tastes and consistencies of food (I totally agree / Partially agree / Indifferent / Partially disagree / Totally disagree)
   4. BLW helps mothers simplify food introduction (I totally agree / Partially agree / Indifferent / Partially disagree / Totally disagree)
   5. BLW enhances chewing instead of sucking (I totally agree / Partially agree / Indifferent / Partially disagree / Totally disagree)
   6. BLW promotes the development of fine motor skills (I totally agree / Partially agree / Indifferent / Partially disagree / Totally disagree)
   7. BLW can prevent obesity (I totally agree / Partially agree / Indifferent / Partially disagree / Totally disagree)
   8. BLW can prevent food-related conflicts (I totally agree / Partially agree / Indifferent / Partially disagree / Totally disagree)
   9. BLW babies won't gain enough weight (I totally agree / Partially agree / Indifferent / Partially disagree / Totally disagree)
   10. BLW can contribute to a lack of certain nutrients (I totally agree / Partially agree / Indifferent / Partially disagree / Totally disagree)
   11. BLW can contribute to children's development (I totally agree / Partially agree / Indifferent / Partially disagree / Totally disagree)
   12. BLW can be very convenient, as there is no need to prepare special food for children (as it simplifies food preparation) (I totally agree / Partially agree / Indifferent / Partially disagree / Totally disagree)

Socio-demographic data

1. Sex
   1. Female
   2. Male
2. Age
3. Length of professional experience (in years)
4. What is your professional category?
   1. Nurse
   2. Nutritionist / Dietitian
   3. Other health professional
5. Indicate the highest level of academic qualification
